# Coordination-Driven Poly[2]Pseudorotaxanes in Highly Polar Organic Solvent

**DOI:** 10.3389/fchem.2020.00579

**Published:** 2020-07-30

**Authors:** Hang Su, Wei Chen, Liang Li, Bin Li, Zhi-Yuan Zhang, Chunju Li

**Affiliations:** ^1^School of Chemical and Environmental Engineering, Shanghai Institute of Technology, Shanghai, China; ^2^Department of Chemistry, Center for Supramolecular Chemistry and Catalysis, Shanghai University, Shanghai, China; ^3^Tianjin Key Laboratory of Structure and Performance for Functional Molecules, College of Chemistry, Tianjin Normal University, Tianjin, China

**Keywords:** pillararenes, host-guest interactions, coordination polymers, polypseudorotaxanes, supramolecular chemistry

## Abstract

Self-assembly of polypseudorotaxanes in high-polar organic solvents is difficult due to remarkably weak interactions between macrocycles and axles. Reported here is a novel metal-coordinated poly[2]pseudorotaxane constructed by pillar[5]arene, 1,4-bis(4-pyridyl pyridinium)butane, and [PdCl_2_(PhCN)_2_] in highly polar organic solvent of dimethyl sulfoxide (DMSO). Utilizing a combination of ^1^H NMR, NOESY, DOSY, DLS, SEM, and viscosity measurements, the formation of polypseudorotaxane was shown to be dependent on the concentration of [2]pseudorotaxanes/[PdCl_2_(PhCN)_2_] and temperature. Furthermore, a temperature-responsive supramolecular gel with reversibly gel–sol transformation was obtained via spontaneous assembly of the polypseudorotaxanes at high concentrations.

## Introduction

Over the past 20 years, supramolecular architectures of (pseudo)rotaxanes and catenanes have played a significant role in supramolecular topology and the fabrication of mechanically interlocked molecules (Loeb, 2007; Serreli et al., 2007; Hunter, 2011; Lehn, 2017). Poly(pseudo)rotaxanes constructed by threading repeated macrocyclic rings onto linear-chain polymeric backbones have attracted tremendous attention for their specific and unique molecular recognition structures and diverse potential applications in various fields (Forgan et al., 2011; Du et al., 2012; Rambo et al., 2012; Rotzler and Mayor, 2013; Guo and Liu, 2014; Ma and Tian, 2014; Hou et al., 2016; Lefebvre et al., 2016; Kato et al., 2018; Hashidzume et al., 2019; Xiao et al., 2020).

Macrocycles are the basic building blocks in the construction of pseudorotaxanes because of the strong binding ability between macrocyclic hosts and guests. Therefore, there is no doubt that introducing new macrocycles and novel non-covalent interactions into polypseudorotaxanes will expand the applications of polypseudorotaxanes. Furthermore, variations in supramolecular structures allow them to show unique responsivity to stimuli. Pillar[n]arenes, the fifth generation of host macrocycles, have been applied to the formation of various functional supramolecular materials, owing to their rigid pillar architecture, easy functionalization, and outstanding binding properties in host–guest chemistry (Cao et al., 2009; Xue et al., 2012; Li, 2014; Ogoshi et al., 2016; Li et al., 2017; Hua et al., 2018, 2019; Chen et al., 2019; Xia et al., 2019; Shao et al., 2020; Wang et al., 2020). To date, a variety of supramolecular poly(pseudo)rotaxanes based on pillar[n]arenes have been investigated (Hu et al., 2012; Eichstaedt et al., 2016; Cui et al., 2017; Zeng et al., 2018; Li B. et al., 2019; Yang et al., 2019).

Metal coordination interactions, as a class of non-covalent interactions possessing remarkable stability and unique properties, can be used to effectively and conveniently generate polypseudorotaxane (Lee et al., 2001; Liu et al., 2003; Harada et al., 2009; Wei et al., 2014; Yan et al., 2014; Krogsgaard et al., 2016; Tian et al., 2016; Winter and Schubert, 2016; Wu et al., 2016; Huang et al., 2017; Wang et al., 2018 Xia et al., 2018; Wang L. et al., 2019; Zhu et al., 2019). However, most of the (poly)rotaxanes and (poly)pseudorotaxanes are constructed in water, low polar organic solvents, or the crystalline state. Highly polar organic solvent such as dimethyl sulfoxide (DMSO) seems to be not working because, generally, the non-covalent interactions between the wheels and axles, which greatly depend on the sorts and polarity of solvents, are quite weak in DMSO. Aqueous solution and low polar organic solvents can maintain these non-covalent interactions well. But highly polar solvents such as DMSO inhibit non-covalent bonds involving hydrogen bonding and complementary π···π-stacking, through powerful solvation of the interacting components.

**Scheme 1:**
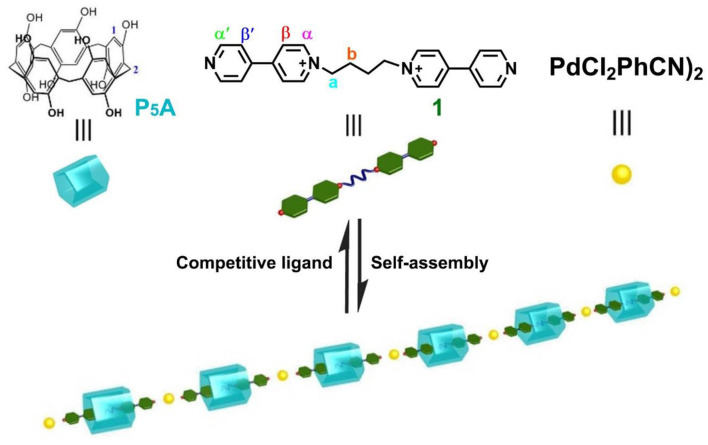
Schematic representation of the conversion from [2]pseudorotaxanes to linear polypseudorotaxane.

In the past 10 years, our group focused on the host–guest chemistry of pillararenes and biphenarenes (Li, 2014; Ma et al., 2016; Li H. et al., 2019; Wang Y. et al., 2019; Xu et al., 2020). The association constant, (7.4 ± 0.3) × 10^2^ M^−1^, of **P**_**5**_**A** and bis(pyridinium)dicationic guest in DMSO is surprisingly high, leading to the formation of a [2]psdudorotaxane-type complex (Li et al., 2010). Herein, to provide new insight into supramolecular polypseudorotaxanes in highly polar solvents, we extended our research target to novel P_5_A-based polypseudorotaxane bridging by palladium(II)-containing coordination interactions [PdCl_2_(PhCN)_2_]. Therefore, a linear polypseudorotaxane was constructed by [2]pseudorotaxanes making up of **P**_**5**_**A** and bis(pyridinium)dicationic (**1**) via metal–coordination interactions in DMSO (Scheme 1). It was expected that the utilization of P_5_A-based [2]pseudorotaxanes and metal–ligand coordination would be quite suitable for fabricating polymeric assemblies in highly polar solvents due to their robust interactions. Interestingly, the obtained polypseudorotaxane could continuously self-assemble at higher concentrations to form a dynamic supramolecular gel, which responded to environmental stimuli.

## Materials and Methods

All reagents and solvents were commercially available and used without further purification, unless otherwise noted. Compound (**P**_**5**_**A**) (Ogoshi et al., 2008; Cao et al., 2009) and bis(pyridinium)dicationic **1** (Joseph et al., 2003; Li et al., 2010) were synthesized according to literature procedures. ^1^H NMR and DOSY spectra were recorded on a Bruker AV500 instrument. Viscosity measurements were carried out with Ubbelohde micro dilution viscometers (Shanghai Liangjing Glass Instrument Factory, 0.40 mm inner diameter) at 298 K in DMSO. Dynamic light scattering (DLS) was analyzed on a Malvern Zetasizer 3000HSA at 298 K. Scanning electron microscopy (SEM) images were recorded on SHIMADZU SSX-550.

## Results and Discussion

**Figure 1:**
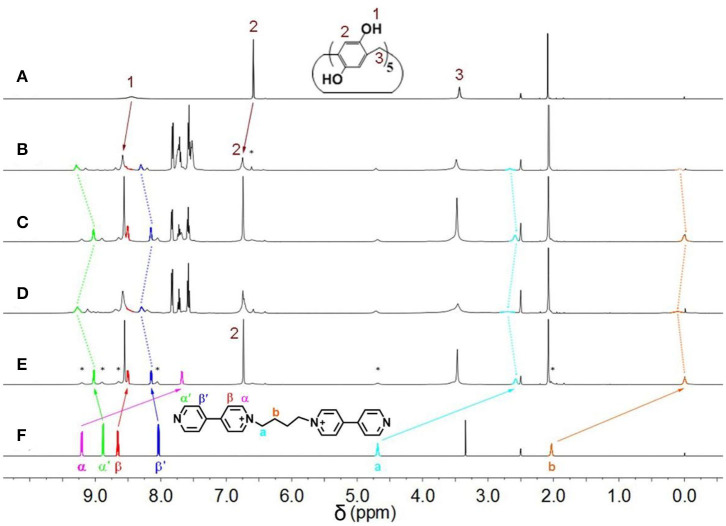
^1^H NMR spectra (DMSO-*d*_6_, 298 K, 500 MHz) of polypseudorotaxane and building units. **(A) P**_**5**_**A**; **(B) P**_**5**_**A** + **1** **+** [PdCl_2_(PhCN)_2_] + PPh_3_ + [PdCl_2_(PhCN)_2_]; **(C) P**_**5**_**A** + **1** **+** [PdCl_2_(PhCN)_2_] + PPh_3_; **(D) P**_**5**_**A** + **1** **+** [PdCl_2_(PhCN)_2_]; **(E) P**_**5**_**A** + **1**, **(F) 1**. ([**P**_**5**_**A**] = [**1**] = [PdCl_2_(PhCN)_2_] = 60.0 mM; *represent uncomplexed **P**_**5**_**A** and free **1**).

**Figure 2:**
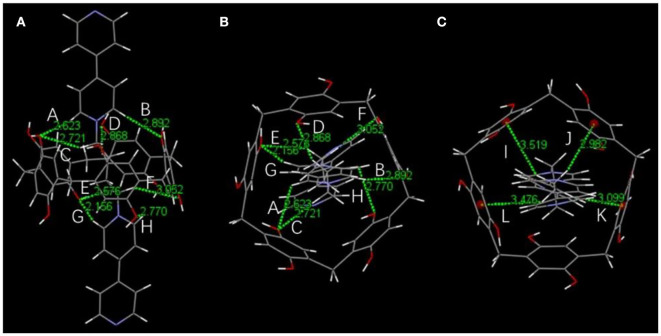
The energy-minimized structure of [2]pseudorotaxanes calculated by DFT (Materials Studio). The green dashed lines represent hydrogen bond interactions (A–H) and C–H…π interactions (I–L). **(A)** Front view and **(B)** top view of hydrogen bond parameters: H…O distance (Å), C(O)–H…O angle (°) A: 2.623, 122.75; B: 2.892, 169,46; C: 2.721, 147.14; D: 2.868, 144.38; E: 2.576, 144.90; F: 3.052, 148.60; G: 2.156, 161.67; H: 2.770, 101.37. **(C)** Top view of C–H…π interaction parameters: C–H…π distance (Å), C–H…π angle (°) I: 3.519, 138.60; J: 2.982, 156.63; K: 3.099, 142.02; L: 3.476, 133.24.

Initially, host–guest complexation of **P**_**5**_**A** and **1** was carried out in DMSO-*d*_6_ and investigated through ^1^H NMR spectroscopy. As shown in Figures 1A,E,F, the ^1^H NMR spectra of **1** were recorded in the absence and presence of the **P**_**5**_**A** host, where evident upfield chemical shifts and a broadening effect inside the pyridine motif and methylene protons (H_a_, H_b_, H_α_, and H_β_) on guest **1** could be observed in the presence of **P**_**5**_**A** owing to the shielding effects in the cavity, while no apparent change was observed in the proton signals of ${\text{H}}_{{\text{α}}^{\prime }}$ and ${\text{H}}_{{\text{β}}^{\prime }}$ on guest **1**. When comparing to the corresponding signals of the uncomplexed **P**_**5**_**A** and **1**, new peaks were observed, demonstrating a slow exchange on the NMR timescale for this binding process. The results are in agreement with the spatial structure that the host **P**_**5**_**A** as a wheel was fully threaded by the axle of guest **1** and left pyridine moiety outside its cavity, indicating the formation of a [2]pseudorotaxane between **P**_**5**_**A** and **1** in DMSO (Scheme 1). Besides, distinct NOE correlation signals between the protons H_a_, H_b_, and H_α_ on **1** and H_1−3_ on **P**_**5**_**A** in a 2D NOESY spectrum further confirmed the formation of the [2]pseudorotaxanes (Supplementary Figure 1). As shown in the energy-minimized structure of the [2]pseudorotaxanes calculated by DFT (Materials Studio), multiple hydrogen bonding and C–H…π interactions between **P**_**5**_**A** and **1** provided enough non-covalent interactions and guaranteed the existence of [2]pseudorotaxanes in DMSO. And the pyridine moiety on **1** was located outside the cavity of **P**_**5**_**A**, which reserved indispensable sites for the coordination of metal (Figure 2).

**Figure 3:**
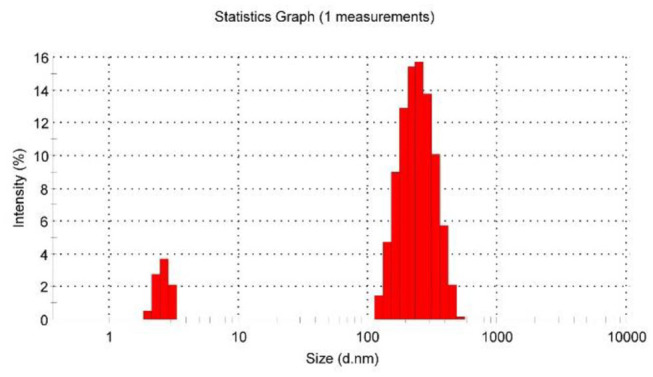
Dynamic light scattering (DLS) spectrum of polypseudorotaxane in DMSO ([PdCl_2_(PhCN)_2_]= [2]pseudorotaxanes = 60 mM, 298K).

Subsequently, after dissolving 1.0 equiv of [PdCl_2_(PhCN)_2_] into 60 mM premixed solution of **P**_**5**_**A** and **1**, all of the main peaks broadened remarkably, and the signals of ${\text{H}}_{{\text{α}}^{\prime }}$ and ${\text{H}}_{{\text{β}}^{\prime }}$ on guest **1** clearly shifted downfield (Figure 1D). These observations provided clear evidence of the complexation between pyridine nitrogen atoms and palladium(II) ligands. Furthermore, binding stoichiometry between [PdCl_2_(PhCN)_2_] and **1** was investigated. To a mixture of **1** and **P**_**5**_**A** ([**1**]: [**P**_**5**_**A**] = 1:5) in DMSO-*d*_6_, [PdCl_2_(PhCN)_2_] was added in different ratios and ^1^H NMR spectra were recorded. As shown in Supplementary Figure 2, upon increasing [PdCl_2_(PhCN)_2_], both the proton signals of ${\text{H}}_{{\text{α}}^{\prime }}$ and ${\text{H}}_{{\text{β}}^{\prime }}$ on the pyridine rings of **1** shifted downfield significantly, suggesting the coordination of metal to the pyridine rings. No obvious change was observed for the signals of ${\text{H}}_{{\text{α}}^{\prime }}$ and ${\text{H}}_{{\text{β}}^{\prime }}$ when the molar ratio of [**1**]: [PdCl_2_(PhCN)_2_] was increased to 1:1, indicating that the binding ratio between [PdCl_2_(PhCN)_2_] and **1** was 1:1 or n:n, which fitted well with the coordination characteristics between pyridine and [PdCl_2_(PhCN)_2_] (Kaminker et al., 2011). The 255 nm of the hydrodynamic radius measured by dynamic light scattering (DLS) manifested the formation of large aggregates, which excluded the 1:1 mode and confirmed that the binding ratio was n:n. The small amount of specie in several nanometers was deduced as unreacted [2]pseudorotaxanes (Figure 3). These results verified the formation of metal supramolecular polypseudorotaxane between [PdCl_2_(PhCN)_2_] and [2]pseudorotaxane.

Furthermore, the competitive ligand PPh_3_ was employed to bind palladium(II) ions to investigate the phase transition between polypseudorotaxane and [2]pseudorotaxane. Upon adding **1** equiv of PPh_3_ to the polypseudorotaxane, a precipitate formed at the bottom of the mixed system solution. As shown in Figure 1C, the ^1^H NMR spectrum was almost the same with the spectrum of [2]pseudorotaxane (Figure 1E), which indicated the formation of the more stable complex between PPh_3_ and palladium(II) ions, resulting in the disassembly of the polypseudorotaxane (Wang et al., 2010). After filtrating off the precipitate, one equiv of [PdCl_2_(PhCN)_2_] was added to the solution, and the peaks of protons on guest **1** returned to their original positions (Figure 1B). This result suggested that metallosupramolecular polypseudorotaxane was reconstructed. Therefore, the reversible transition between polypseudorotaxane and [2]pseudorotaxane can be realized.

**Figure 4:**
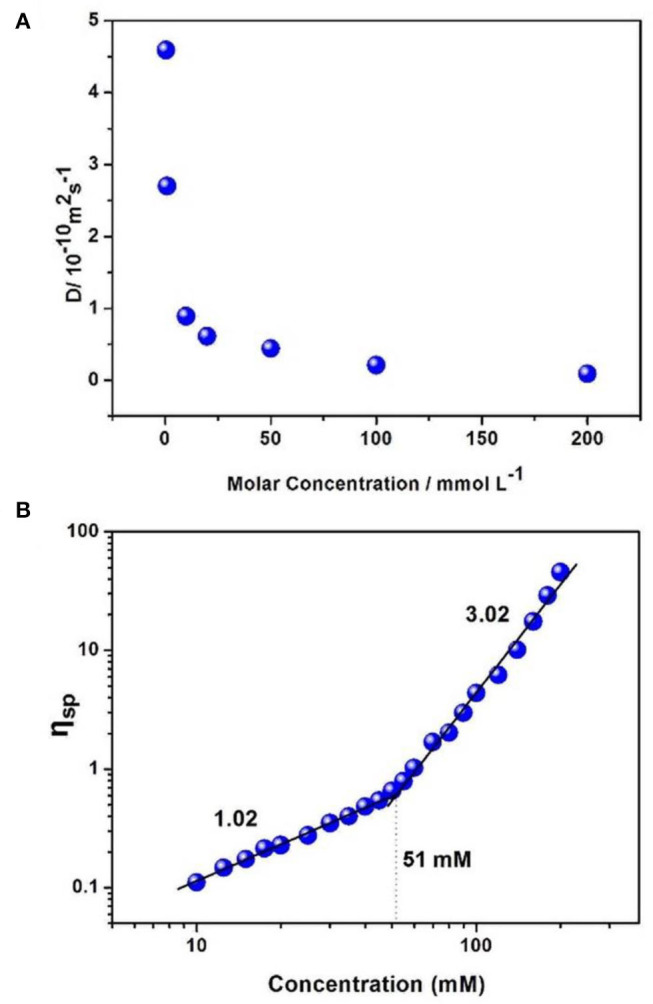
**(A)** Concentration dependence of diffusion coefficient D (500 MHz, CDCl_3_, 298 K) of [PdCl_2_(PhCN)_2_] and [2]pseudorotaxanes in a 1:1 molar ratio. **(B)** Specific viscosity of equimolar mixtures of [2]pseudorotaxanes and [PdCl_2_(PhCN)_2_] vs. the [PdCl_2_(PhCN)_2_]/[2]pseudorotaxanes concentration (DMSO, 298 K).

Two-dimensional diffusion-ordered NMR experiments (DOSY) were adopted to explore the polypseudorotaxane. When the concentration of [2]pseudorotaxane/[PdCl_2_(PhCN)_2_] increased from 0.5 to 200 mM, the weight average diffusion coefficient (D) decreased significantly from 4.59 × 10^−10^ to 0.88 × 10^−11^ m^2^ s^−1^, suggesting an increase in the average size of the polymeric structure owing to the generation of polypseudorotaxane from the small oligomers (Figure 4A).

Viscosity is a characteristic property index for metallosupramolecular polypseudorotaxane. Therefore, viscosity measurements of an equimolar mixture of [2]pseudorotaxanes and [PdCl_2_(PhCN)_2_] were carried out in DMSO at 298 K. A double-logarithmic plot of specific viscosity vs. the initial concentrations of [2]pseudorotaxanes is shown in Figure 4B. The slopes of the curves in the low-concentration region tended to 1 (1.02 for [PdCl_2_(PhCN)_2_]/[2]pseudorotaxanes), implying that no linear polypseudorotaxane formed (Söntjens et al., 2001; Xiao et al., 2012). When the concentrations of the mixture of [2]pseudorotaxanes and [PdCl_2_(PhCN)_2_] increased above the critical polymerization concentration (CPC, approximately 51 mM), a sharp increase in the viscosity was obtained (slope = 3.02), which indicated the formation of linear polypseudorotaxane resulting from strong interactions between [2]pseudorotaxanes and [PdCl_2_(PhCN)_2_] (Söntjens et al., 2001; Xiao et al., 2012). This result is also in agreement with the above NMR experiments.

**Figure 5:**
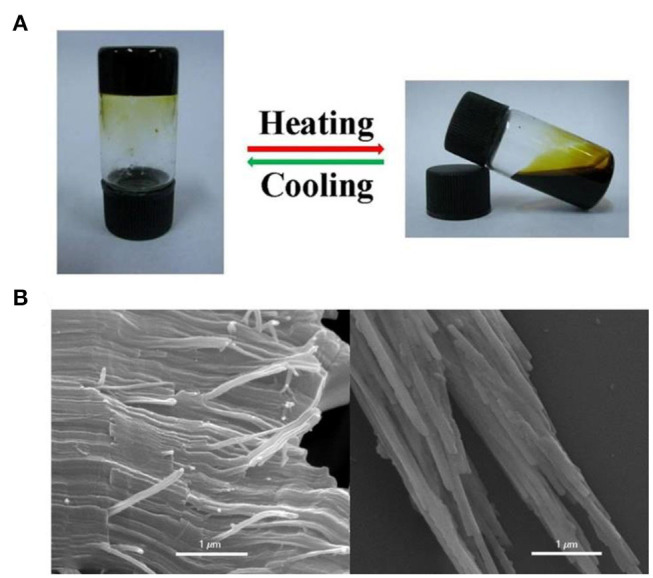
**(A)** The reversible gel–sol transformation of the polypseudorotaxane gel (500 mM) induced by temperature. **(B)** SEM images of the supramolecular xerogels.

Interestingly, when the concentration exceeded 500 mM, a cross-linked supramolecular gel was observed. That was, upon increasing the concentration of [2]pseudorotaxane and [PdCl_2_(PhCN)_2_], metallosupramolecular polypseudorotaxane transformed into a supramolecular gel. Notably, the metal-coordinated polypseudorotaxane gel was sensitive to temperature and could transform into sol reversibly by heating to 60°C and cooling to room temperature (25°C) (Figure 5A). A possible reason for the reversible gel–sol transition is reversible entanglement among linear polypseudorotaxane and the coordination interaction between [2]pseudorotaxane and [PdCl_2_(PhCN)_2_]. Heating decreased that interaction and decomposed the polypseudorotaxane, and therefore, gel changed to sol. However, upon cooling, the intermolecular entanglement restored, resulting in the recovery of the supramolecular gel. The morphology of polypseudorotaxane xerogels prepared by freeze-drying was investigated by scanning electron microscopy (SEM). Regular long and fine fiber structures were observed, and the diameter was determined to be 0.2–0.3 μm (Figure 5B). These observations provided further proof that the metallosupramolecular gel was constructed by polypseudorotaxane fibers resulting from [2]pseudorotaxanes and bridging palladium(II).

## Conclusions

In summary, a novel metallosupramolecular polypseudorotaxane was successfully fabricated from pillar[5]arene-based [2]pseudorotaxanes and [PdCl_2_(PhCN)_2_] in a highly polar solvent of DMSO, which was comprehensively confirmed by various techniques, such as ^1^H NMR, NOESY, DOSY, DLS, Viscometry, and SEM. The formation of polypseudorotaxane was shown to be dependent on the concentration of [2]pseudorotaxanes/[PdCl_2_(PhCN)_2_] and temperature. Moreover, the reversal transition between polypseudorotaxane and [2]pseudorotaxanes can be realized by the successive addition of metal linker [PdCl_2_(PhCN)_2_] and competitive ligand PPh_3_. Significantly, the metal polypseudorotaxane could transform into supramolecular gel when the concentration was above 500 mM, which showed reversibly temperature-induced gel–sol transformation. This study provides a new insight into the construction of macrocycles-based polypseudorotaxane in highly polar organic solvent and benefits to the fabrication of smart materials.

## Data Availability Statement

The raw data supporting the conclusions of this article will be made available by the authors, without undue reservation.

## Author Contributions

CL, BL, and LL conceived of this project and designed the experiments. HS and WC contributed to most of the experimental work. All authors analyzed the data. CL, WC, and Z-YZ co-wrote the paper. All authors discussed and commented on the paper.

## Conflict of Interest

The authors declare that the research was conducted in the absence of any commercial or financial relationships that could be construed as a potential conflict of interest.
