# Supplementary material for: Coordination-Driven Poly[2]Pseudorotaxanes in Highly Polar Organic Solvent
Source: Front Chem. 2020 Jul 30;8:579. doi: 10.3389/fchem.2020.00579 (PMC7406859; doi:10.3389/fchem.2020.00579)
Supplement: Supplementary file 1 [file Data_Sheet_1.PDF]

Supplementary Material

Supplementary Figures

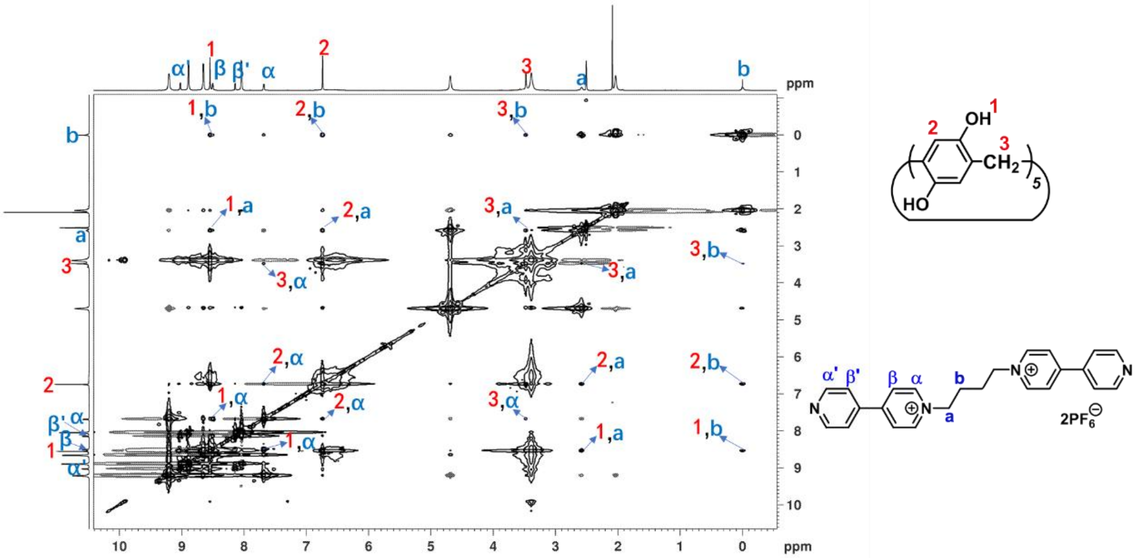

Supplementary Figure 1. 2D NOESY spectrum of $P_5A \supset 1$ (400 MHz, DMSO-d_6, 298 K).

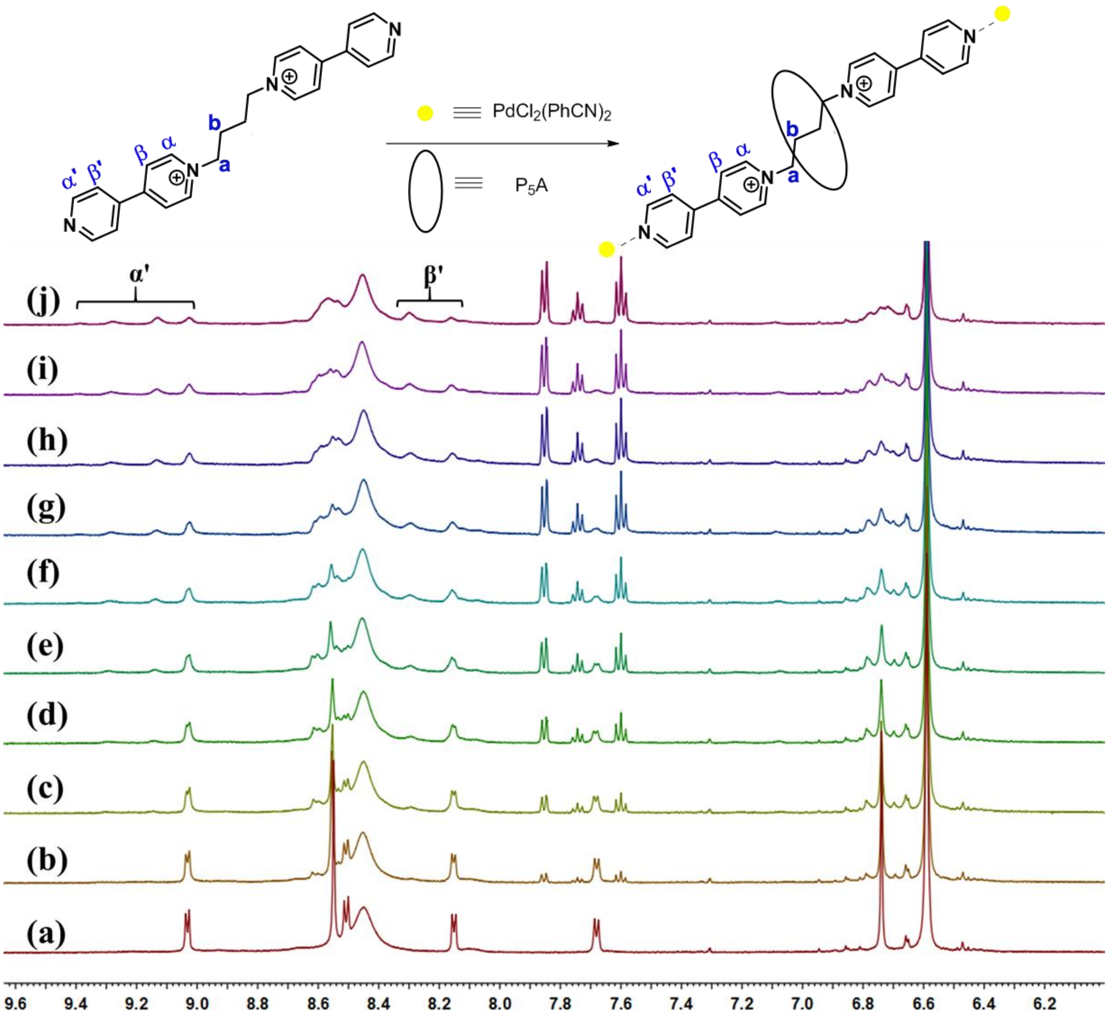

Supplementary Figure 2. ^{1}H NMR spectra (400 MHz, 298 K) of the mixed solution of **P$_5$A** and **1** ([**1**] = 4 mM, [P$_5$A] = 20 mM) in DMSO-d_6 with varying molar ratios of [PdCl$_2$(PhCN)$_2$]: (a) 0 mM, (b) 0.65 mM, (c) 1.26 mM (d) 1.87 mM, (e) 2.48 mM, (f) 3.08 mM, (g) 3.66 mM, (h) 3.95 mM, (i) 4.24 mM, and (j) 5.37mM.
